# Randomized clinical trial on the use of a colon-occlusion device to assist rectal washout

**DOI:** 10.1007/s00464-020-07992-9

**Published:** 2020-09-23

**Authors:** Carolin Cordewener, Manuel Zürcher, Philip C. Müller, Beat P. Müller-Stich, Andreas Zerz, Georg R. Linke, Daniel C. Steinemann

**Affiliations:** 1 Pelvic Floor Unit, Clarunis, University Center for Gastrointestinal and Liver Diseases, 4002 Basel, Switzerland; 2 Department of Surgery, Spital Thun STS AG, Krankenhausstrasse 12, 3600 Thun, Switzerland; 3 Department of Visceral and Transplantation Surgery, University Hospital Zürich, Rämistrasse 100, 8032 Zurich, Switzerland; 4 Department of Surgery, University Hospital Heidelberg, Im Neuenheimer Feld 110, 69120 Heidelberg, Germany; 5 eSwiss Medical and Surgical Center, Klinik Stephanshorn, Brauerstrasse 97, 9016 St. Gallen, Switzerland; 6 Medical Faculty, University Basel, Klingelbergstrasse 61, 4056 Basel, Switzerland

**Keywords:** Colon occlusion, Natural orifice transluminal endoscopic surgery, Transrectal, Peritoneal contamination, Proctology

## Abstract

**Background:**

Transrectal Natural Orifice Transluminal Endoscopic Surgery is currently limited by the inherent risk of surgical site infection due to peritoneal contamination after rectotomy. Coloshield has been developed as a temporary colon occlusion device to facilitate rectal washout. However, effectiveness and safety has not been evaluated in humans.

**Methods:**

Twenty-two patients have been randomly assigned to undergo proctological intervention with a rectal washout with and without the use of Coloshield. Patients and assessors were blinded. Boston Bowel Preparation Scale (BBPS) has been determined 30 min as well as immediately after rectal washout. Feasibility, pain, intra- and postoperative morbidity as well as bowel function and continence 6 weeks after surgery were assessed.

**Results:**

BBPS 30 min after rectal washout with and without Coloshield was in mean 2.42 ± 1.02 and 2.12 ± 0.89 (*p* = 0.042). Mean BBPS immediately after rectal washout was 2.39 ± 1.02 and 2.24 ± 0.66 (*p* = 0.269). Mean BBPS immediately after rectal washout and 30 min thereafter did not differ (*p* = 0.711). Coloshield application was feasible without any complications. The median (interquartile range) numeric rating scale for pain 4 h after surgery was 1 (0–1) and 3 (0–4) (*p* = 0.212). Six weeks after surgery 0/11 and 1/11 patients suffered from evacuation difficulties (*p* = 1.0) and the median Vaizey–Wexner score was 1 (0–3) and 1 (0–2) (*p* = 0.360).

**Conclusions:**

Coloshield application in humans is feasible and safe. Slight benefits in rectal preparation by washout are found when Coloshield is used. Colon occlusion by Coloshield for transrectal NOTES should be evaluated within clinical studies.

**Trial registration:**

Clinicaltrials.gov NCT02579330

In procedures that involve a rectotomy such as transanal endoscopic microsurgery (TEM) or transrectal natural orifice transluminal endoscopic surgery (NOTES) there is an inherent risk of pelvic or abdominal sepsis and of leakage from insufficiency of a rectotomy closure. In TEM suture line dehiscence after TEM is described to be as frequent as 23% in chemoradiation-naïve rectum [1]. However, dehiscence in the extraperitoneal part of the rectum does not necessarily translate into pelvic sepsis considering the ability of the well vascularized mesorectal fat to serve as a matrix for rectal wall regrowth. In a series of 262 TEM procedures 2.7% of the patients developed pelvic sepsis despite an orthograde lavage and single shot antibiotic prophylaxis was performed [2]. Therefore, even open wound management after TEM has been proposed with similar results compared to suturing the defect [3].

Hybrid NOTES represents an alternative to laparoscopic surgery for several routine procedures [4]. Hybrid NOTES is safe and reduces postoperative pain [5]. Most accepted routes for NOTES are the transvaginal access followed by the transgastric access. However, the transrectal route is only used in left colonic or rectal resections when the colon or rectums needs to be divided for the procedure [6]. Surgical procedures that are remote from the rectum, in which a rectotomy would be needed to use the transrectal route, are currently only used in experimental animal models [7–9]. One of the limitations for the use of the transrectal access is the contamination of the colorectum that may lead to peritoneal septic complications. Even after mechanical bowel preparation positive abdominal swabs were obtained in left colonic resections with transrectal natural orifice specimen extraction in up to 100% of patients [10, 11]. Therefore usually orthograde mechanical bowel preparation is performed in combination with a rectal washout [12]. However, mechanical bowel preparation is not well tolerated by patients and it has been shown that contamination of the rectal mucosa remains high even after extensive rectal washout [13, 14].

Measurements to prepare the rectal mucosa and reduce the bacterial load, however, are included in the perioperative management for TEM and transrectal NOTES. This involves orthograde bowel preparation, oral antibiotic prophylaxis, and intravenous antibiotic prophylaxis. Although comparative clinical studies are missing rectal washout is commonly performed [15]. Therefore, a colon occlusion device has been developed enabling a temporary mechanical occlusion of the colon during the intervention. Recontamination after rectal washout by passage of liquid stool from the oral colon is avoided. A novel device called Coloshield has been designed. Coloshield consists of a catheter with a double balloon that can be inflated to block the colon. Between the balloons there is a negative pressure zone preventing dislocation of the catheter. The colon occlusion device enables the possibility for rectal washout and maintains a clear operating field during surgery. In a porcine study safety and feasibility of Coloshield was demonstrated [16], peritoneal contamination during transrectal NOTES procedures was significantly reduced [17]. By the use of an optimized rectal washout in combination with Coloshield peritoneal contamination in transrectal NOTES was even avoided entirely [14]. Nevertheless, effectiveness and safety of Coloshield has not been evaluated in humans so far.

This randomized clinical study aimed, therefore, at comparing the macroscopic rectal bowel preparation with and without use of Coloshield after a rectal washout in patients undergoing proctologic interventions.

## Materials and methods

This was a prospective, randomized clinical, single-center trial comparing the effectiveness and safety of a rectal washout with and without the use of Coloshield in a group of patients undergoing surgery for fistula-in-ano, or hemorrhoids. The study is reported according to the CONSORT statement. The exclusion criteria were pregnancy, emergency operation, rectal stricture or stenosis, status post rectal resection or pelvic radiation therapy, any inflammatory bowel disease with inclusion of the rectum as well as the need for mechanical bowel preparation. Patients included in this study provided informed consent and were aged > 18 years. The Ethical Committee of Northwestern Switzerland and the Cantonal Ethical Committee of Berne approved this study (EKNZ 2015-341/PB_2016-02478). The study was registered in clinicaltrials.gov (NCT02579330). The procedures were performed at the Hospital of Thun, Switzerland.

All patients that met the inclusion criteria were invited to participate in the study, which lasted for six weeks, and were randomized by online based randomization software (sealedenvelope.com) [18]. The randomization was undertaken prior to surgery while the patient was in the operating room. Requested by the patient, the anesthesia was either general or spinal. Patient and assessors were blinded for the allocation to the study or control group. However, the surgeon was aware of this fact. Data from the enrolled patients were prospectively included in an institutional study registry which was based on case reporting forms and an excel spreadsheet. This included demographic data such as age, sex, indication for surgery, numeric rating scale (NRS) for pain before surgery, the anorectal function before surgery, as well as clinical data such as duration of surgery, NRS for pain 4 h, 24 h, 48 h, and 72 h after surgery. Bowel habits such as bowel movements per day, evacuation difficulties, and intake of laxatives, stool consistence, and the Vaizey–Wexner score [19] were recorded preoperatively and after 6 weeks. To collect postoperative outpatient data each patient received a pain diary to document the intensity of pain on NRS.

**Fig. 1:**
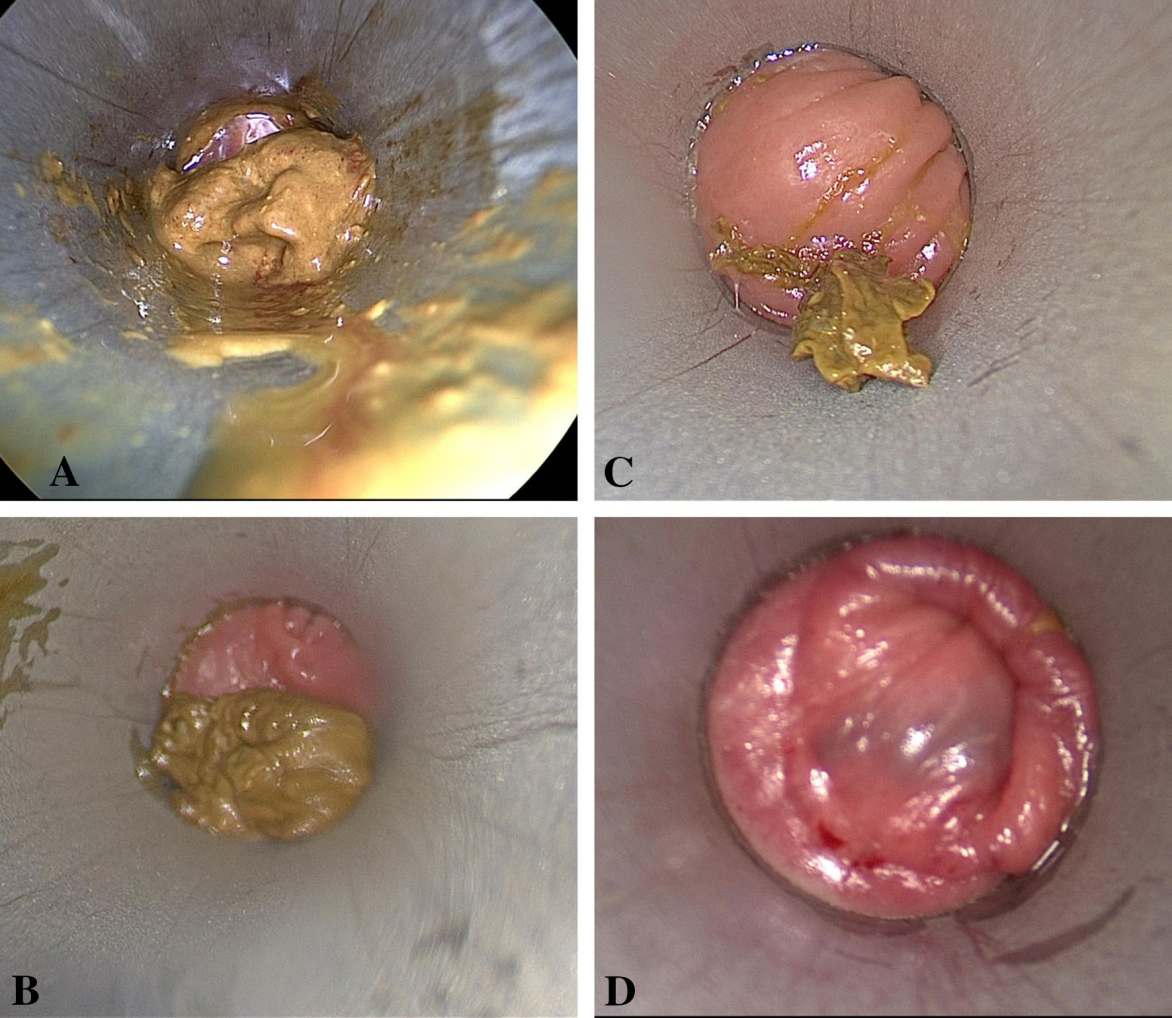
Examples of photographs of the rectal wall with **A** Bowel Preperation Scale (BBPS) of 0, **B** BBPS of 1, **C** BBPS of 2, **D** BBPS of 3

The primary outcome was macroscopic cleanness of the rectum based on the Bowel Preparation Scale (BBPS) 30 min after a rectal washout. In modification of the original version of the BBPS [20] the contamination was scored only in the location of the lower rectum. Intraoperative by use of a rigid 30° angle endoscope that was introduced through a rigid rectoscope a photo of anterior wall of the rectum as well of the posterior wall of the rectum were performed before washout, immediately after washout, and 30 min after washout. Care has been taken that the Coloshield device was not visible on the photographs. The photos were rated by three independent assessors (C.C.; G.R.L.; D.C.S.) that were blinded for the group allocations. For each photo (Fig. 1A–D) a BBPS from 0 (unprepared rectum, mucosa cannot be seen) to 3 (No residual staining, small fragments of stool, or opaque fluid) was given. For analysis of the scores the mean scores for the anterior and posterior wall of all three assessors were used for each time point.

Secondary outcomes were feasibility of Coloshield deployment, dislocation of the Coloshield device during surgery, intraoperative morbidity, postoperative morbidity according to the Clavien-Dindo classification [21], pain and bowel function 6 weeks after surgery.

### Coloshield device

**Fig. 2:**
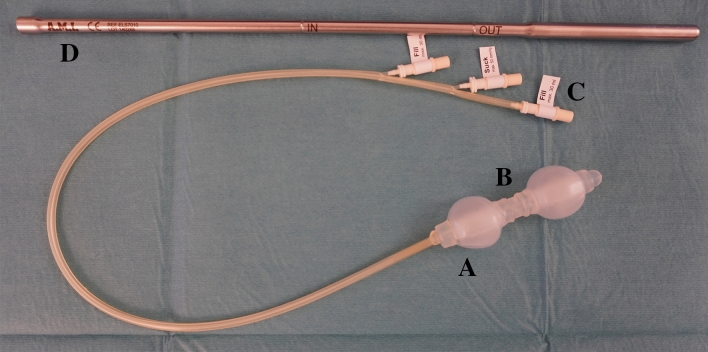
Coloshield with **A** double balloon catheter tip, **B** suction zone, **C** connection tube to fill and suck, and **D** introducer

The principle of Coloshield (certified according to the CE number 0297 (Conformité Européeene), A.M.I., in Feldkirch, Austria) based on two balloons, with negative pressure zone in between. The rod-shaped instrument contained two silicon balloons, which were inflated by means of a catheter. The catheter was diverted at the distal end of the device. Several holes at the section in between the two balloons were connected to another catheter that allowed negative pressure to be established [16] (Fig. 2). By Insufflation of the two balloons and the establishment of a negative pressure between the balloons by means of connection to a vacuum pump the colon was occluded for the duration of surgery.

### Experimental use of the Coloshield device

**Fig. 3:**
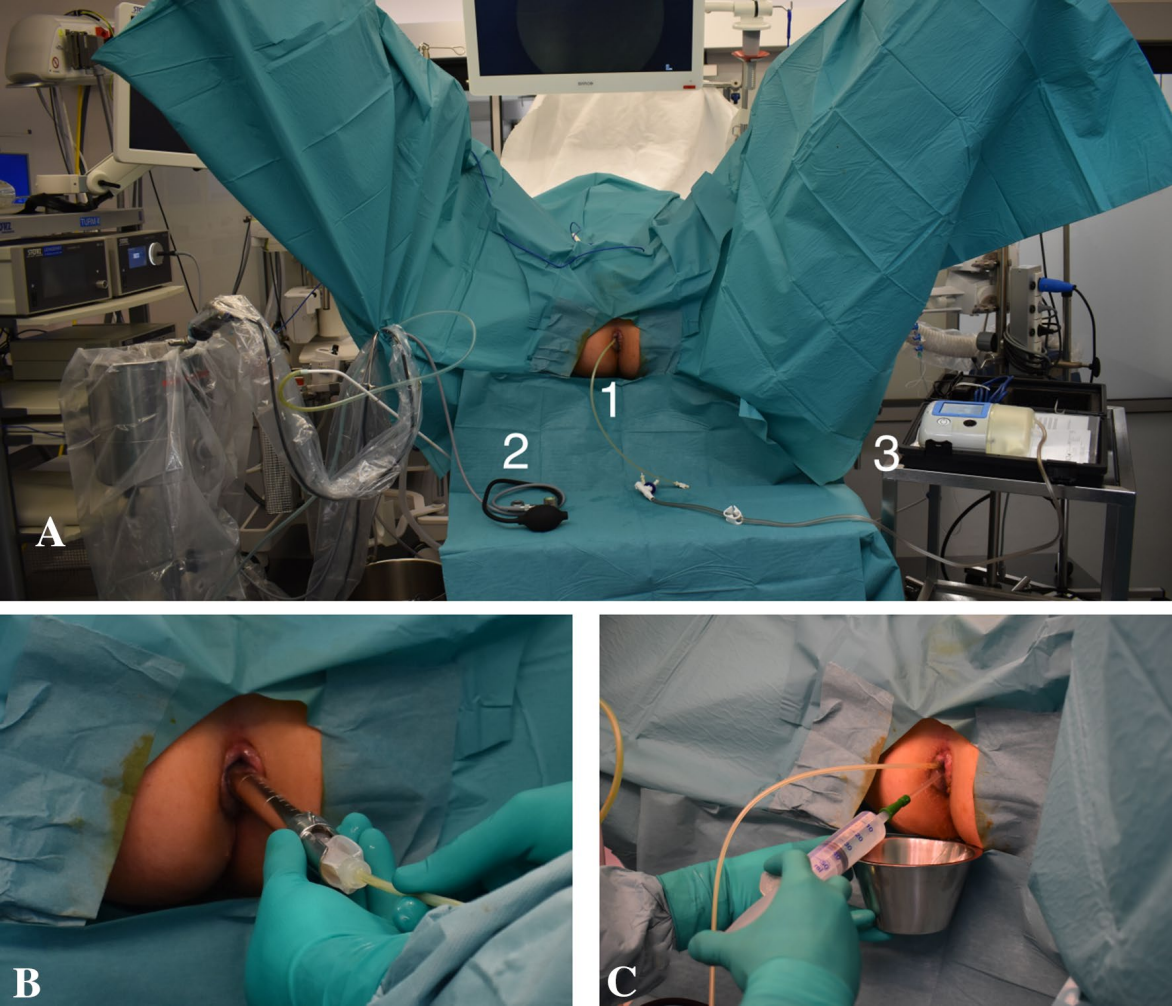
**A** Set-up with **1.** Coloshield inserted, **2.** Rectoscope, and **3.** vacuum pump; **B** Insertion of Coloshield device through rectoscope. **C** Rectal wash-out with saline solution through a rectal tube

No preoperative mechanical bowel preparation or enema was administered preoperatively. After disinfection of the perianal skin with povidone solution and sterile dressing of the operative field a digital rectal examination was performed. A rectoscope was inserted and the integrity of the rectal mucosa was verified. Coloshield was introduced after lubrification with lidocaine Gel. It was positioned 8–10 cm from the anal verge with its aboral end (Fig. 3A–C). Both balloons of the device were inflated with 30 ml air. The second catheter of the device was connected to a vacuum pump with a negative pressure of 50 mmHg. The position of Coloshield in cm from the anal verge was measured by rigid rectoscopy. A rectal washout with 500 ml of saline solution was performed followed by the respective surgical procedure. Thirty minutes after the washout a rectoscopy was repeated to measure again the position of Coloshield in cm from the anal verge. Possible injuries to the rectal mucosa were assessed by rectoscopy. At the end of the surgery the catheter was removed from negative pressure, the balloons were deflated and the device removed.

### Control group

In the control group, no preoperative mechanical bowel preparation or enema was administered. After disinfection of the perianal skin with povidone solution and sterile dressing of the operative field a digital rectal examination was performed. Then a rectal washout with 500 ml of saline solution was performed followed by the respective surgical procedure.

### Sample size calculation and statistical analysis

The study hypothesis is a 30 per cent increase in the intervention group of the BBPS 30 min after washout compared to the control group. To achieve 80% power, with a two-sided *p* < 0.05 taken to show a significant difference, 9 patients per group were required. Analysis was performed on an intention-to-treat basis with all patients included in the group to which they were allocated. With an estimated 20% dropout rate during the follow-up it was planned to include 22 patients in this study with a 1:1 randomization.

Statistical analysis were performed using GraphPad Prism ™ (Version 6 for Mac, GraphPad Software, La Jolla, California, USA). Continuous data was expressed as the mean ± standard deviation or median and interquartile range as indicated. Proportions between groups were compared using a 2-tailed Mann–Whitney test assuming a nonparametric distribution or using an ordinary 1-way ANOVA test for multi-item comparisons. Categorical variables were compared using a 2-sided Fisher’s exact test. Two-sided p values of less than 0.05 were considered to be statistically significant.

## Results

**Fig. 4:**
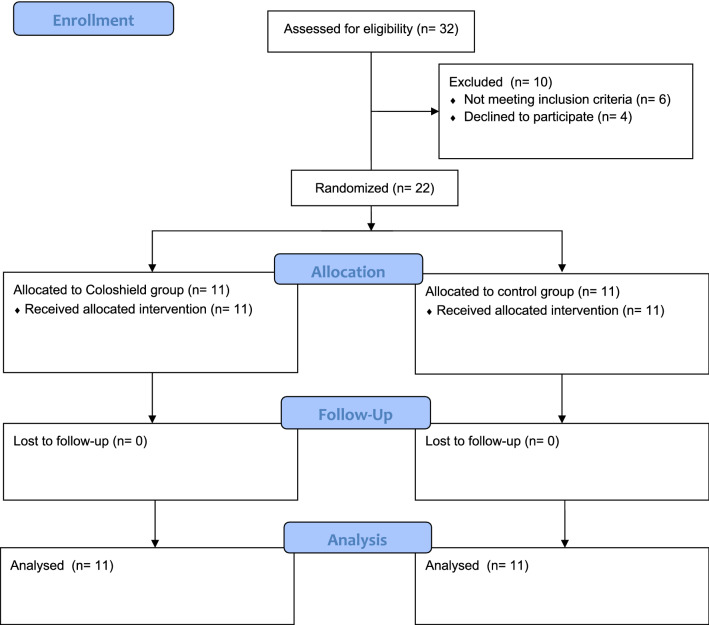
Consort flow diagram

Between January 2019 and September 2019 22 individuals were included (Fig. 4). All randomized patients received the allocated treatment and were analyzed for the primary outcome. Demographic and preoperative data of the Coloshield group and the control group are depicted in Table 1. While there was no difference in the gender distribution among the groups, patients in the control group were younger than patients in the Coloshield group. Anorectal function and the level of incontinence were similar in both groups.

**Table 1:** Demographic and preoperative data for the Coloshield group and the control group

	Coloshield group (*n* = 11)	Control group (*n* = 11)	*p*
Age, median years (IQR)	55 (35–63)	42 (32.5–51.5)	0.031
Gender, female/male	8/3	7/4	1.0
Indication for surgery			
Hemorrhoids	7	8	1.0
Fistula-in-ano	4	3	
Preoperative pain, median NRS (IQR)	1 (0–5)	2 (0–6)	0.938
Daily bowel movements, median (IQR)	1 (1–2)	1.5 (1–2)	0.604
Patients with difficulties to evacuate	4	5	1.0
Patients with use of laxatives	5	2	0.326
Stool consistencea, median points (IQR)	2 (2–2)	2(2–2)	1.0
Vaizey–Wexner Score^b^, median (IQR)	1 (0–3)	1 (0–3)	0.919

IQR = interquartile range; NRS = numeric rating scale (0 = no pain, 10 = maximum pain)

^a^Stool consistence: liquid = 1 point, soft = 2 points, hard = 3 points

^b^Vaizey–Wexner Score: 0 = no incontinence, 24 = maximal incontinence

**Fig. 5:**
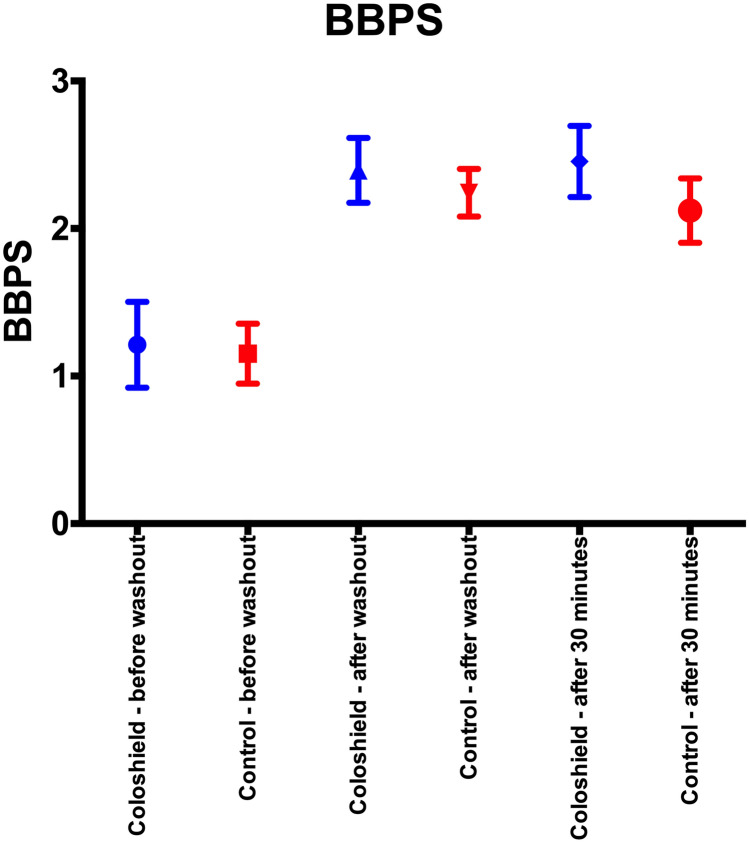
Boston Bowel Preparation Scale in the Coloshield group and the control group before wash-out, after wash-out, and 30 min after wash-out

The macroscopic contamination measured by the BBPS 30 min after rectal washout with and without Coloshield was in mean 2.42 ± 1.02 and 2.12 ± 0.89 (*p* = 0.042; Fig. 5). The mean BBPS immediately after rectal washout was 2.39 ± 1.02 and 2.24 ± 0.66 (*p* = 0.269) in the Coloshield group and control group, respectively. Notably, no re-contamination of the rectum was observed during the 30 min after rectal washout. Mean BBPS immediately after rectal washout and 30 min thereafter did not differ (*p* = 0.711).

Coloshield application was feasible without any complications in all 11 patients. It was possible to deploy the Coloshield successfully in all patients, and besides one patient, the Coloshield stood in place without measurable inadvertent movement. In one patient the Coloshield shifted 3 cm outwards when the vacuum pump was accidentally removed without causing any injuries. In the rectoscopy performed at the end of surgery in one case of the Coloshield group a minor bleeding of the rectal mucosa approximately 3 cm above the dentate line was observed and was self-limiting. It was most likely caused by the anal spreader. The Coloshield device has a separate tube intended to allow rectal washout. However, a clean washout through this tube was not possible due to its narrow diameter of 1 mm. Therefore rectal washout was performed using a rectal catheter through the re-introduced rectoscope.

There was one patient in the Coloshield group that suffered from urinary retention requiring the placement of an indwelling catheter on the day of surgery. This corresponds to a Clavien-Dindo grade II complication. Although there was no postoperative morbidity in the control group the morbidity did not differ (1/11 vs. 0/11; *p* = 1.0). With the exception of the pain level 24 h after surgery that was lower in Coloshield group, the NRS for pain was similar in both groups. Furthermore and importantly, there was no difference in bowel function and fecal continence six weeks after surgery between the Coloshield and control group (Table 2).

**Table 2:** Operative and follow-up data in the Coloshield group and control group

	Coloshield group (*n* = 11)	Control group (*n* = 11)	*p*
Operation time, median minutes (IQR)	33 (32–37	34 (33–38)	0.818
Type of anesthesia, General anesthesia/spinal anesthesia, *n*	10/1	11/0	1.0
Pain on NRS, median (IQR)			
4 h after surgery	1 (0–1)	3 (0–4)	0.212
24 h	1 (0–3.75)	5 (1–8)	0.049
48 h	1.5 (0.25–3.75)	4 (3–7)	0.082
72 h	1 (0.25–5.5)	4 (2–6)	0.374
Daily bowel movements, median (IQR)	1 (1–3)	1.5 (1–2)	1.0
Patients with difficulties to evacuate	0	1	1.0
Patients with use of laxatives	9	6	0.362
Stool consistence^a^, median points (IQR)	2 (2–2)	2(2–2)	0.329
Vaizey–Wexner Score^b^, median (IQR)	1 (0–3)	1 (0–2)	0.360

*IQR* interquartile range, *NRS* numeric rating scale (0 = no pain, 10 = maximum pain)

^a^Stool consistence: liquid = 1 point, soft = 2 points, hard = 3 points

^b^Vaizey–Wexner Score: 0 = no incontinence, 24 = maximal incontinence

## Discussion

In this randomized clinical trial the feasibility of the use of Coloshield in human was demonstrated. The application of Coloshield was safe with no relevant rectal injury and no subsequent postoperative morbidity detected. Moreover, up to six weeks after surgery no deterioration of bowel function or fecal continence was observed. The double balloon catheter allowed an effective occlusion of the upper rectum for at least 30 min. This was demonstrated by a slightly more effective washout compared to the control group and successful maintenance of bowel cleanness during surgery. The difference in BBPS was statistically different but did not reach the presumed clinical difference of 30%.

Coloshield may serve as an effective colon occlusion device during transrectal NOTES. It may contribute towards development of transrectal hybrid-NOTES in abdominal organs that are remote from the rectum such as cholecystectomy [7], prostatectomy [22], nephrectomy [23, 24], or right sided colectomy [25]. Rectal washout with povidone reduced the contamination of the rectal wall and peritoneum in left colic resection (10). It has been shown that peritoneal contamination is massively reduced when Coloshield is used compared to no colon occlusion in a porcine model [17]. Nevertheless, further clinical studies are required to monitor the safety of such approach. Of special concerns are surgical site infections after rectotomy as the rectum will be re-contaminated after extraction of the Coloshield. So, in transanal total mesorectal excision positive pelvic swabs after opening of the rectum are common and do occasionally translate into pelvic surgical site infections [26]. However, in the porcine model in previous studies no infection at the rectotomy site occurred [27, 28]. Although a reduction in contamination is demonstrated (10), further studies are needed to investigate if the clinical infection rate may be reduced by wash out.

The colon occlusion device used in this study has been developed by the current study group [16]. Previously a single balloon catheter for colon occlusion in a porcine model for transrectal peritoneoscopy has been proposed [27]. Furthermore, the use of a detachable occlusion balloon was demonstrated in a porcine model [29]. In the latter experiment balloon dislocation of around 2 cm was described. In a further pig experiment with a detachable colon occlusion balloon transrectal cholecystectomy was performed in nine animals with no occurrence of surgical site infection at necropsy 28 days after surgery [30].

Alternative applications of Coloshield are local rectal resections such as transanal endoscopic microsurgery with the possibility for omission of mechanical bowel preparation [31]. However, the use of Coloshield in proctology would generally not be warranted as a clean operation field is not necessary in such interventions. Proctologic interventions were, however, chosen as a model in the current study as the effectiveness of maintenance of a clean rectum could be safely evaluated when cleanness per se is not mandatory.

The study is limited by its small sample size. However, the sample size calculation demonstrated that the study is powered to demonstrate a considerable high difference in BBPS. The current study is the first trial in human and serves as pilot trial in order to investigate the use of Coloshield mainly in the context of NOTES. The BBPS in both, the Coloshield group and the control group, after rectal washout was suboptimal in some patients. This was mainly owed to the washout being limited to only 500 ml saline solution without the prior application of an enema previously according to the protocol. The administration of a higher amount of saline solution for rectal washout would eventually allow the achievement of higher BBPS. Further studies should also compare colon occlusion by Coloshield with a temporary gauze tamponade. It is important to emphasize that the study focused on the applicability of Coloshield in humans and did not assess the safety of a rectotomy.

In conclusion, Coloshield application in humans is feasible, safe and allows an effective rectal washout that is maintained for at least 30 min. The safety of colon occlusion by Coloshield in transrectal NOTES should be evaluated in further clinical studies.
